# Association between Cognitive Function and Physical Function, Frailty, and Quality of Life in Older Breast Cancer Survivors

**DOI:** 10.3390/cancers16152718

**Published:** 2024-07-31

**Authors:** Diane Von Ah, Carielle Joy Rio, Allie Carter, Susan M. Perkins, Erin Stevens, Ashley Rosko, Ashley Davenport, Mathew Kalady, Anne M. Noonan, Adele Crouch, Susan Storey, Janine Overcash, Claire J. Han, Yesol Yang, Haiying Li, Leorey N. Saligan

**Affiliations:** 1 College of Nursing, The Ohio State University, 394 Newton Hall, 1585 Neil Avenue, Columbus, OH 43210, USAhan.1985@osu.edu (C.J.H.);; 2 Symptoms Biology Unit, Division of Intramural Research, National Institute of Nursing Research, National Institutes of Health, 3 Center Drive, Bethesda, MD 20892, USA; 3 Department of Biostatistics and Health Data Science, IU School of Medicine, Richard M. Fairbanks School of Public Health, Indianapolis, IN 46202, USA; 4 Division of Palliative Care, Department of Internal Medicine, The Ohio State University, 1581 Dodd Drive, 5th Floor North Columbus, Columbus, OH 43210, USA; 5 Division of Hematology, James Comprehensive Cancer Center, The Ohio State University, 1150C Lincoln Tower, Columbus, OH 43210, USA; 6 Division of Medical Oncology, James Comprehensive Cancer Center, The Ohio State University, Columbus, OH 43210, USA; 7 Division of Colon and Rectal Surgery, Clinical Cancer Genetics Program, The Ohio State University-James: Cancer Treatment and Research Center, Columbus, OH 43210, USA; 8 GI Medical Oncology Selection, The Ohio State University-James: Cancer Treatment and Research Center, Columbus, OH 43210, USA; 9 School of Nursing, Indiana University, Indianapolis, IN 46202, USA; adnielse@iu.edu (A.C.); sustorey@iu.edu (S.S.)

**Keywords:** breast cancer, cognitive impairment, physical function, frailty, quality of life, aging, comorbidities

## Abstract

**Simple Summary:**

This study examines the relationship between subjective and objective measures of cognitive function and physical function, frailty, and quality of life (QoL) in older breast cancer survivors. Older breast cancer survivors who reported concerns participated in online surveys that included assessment of patient-reported cognitive function, physical function, frailty, and QoL. Participants then completed objective tests assessing visuospatial working memory and sustained attention. A total of 219 breast cancer survivors completed the study. Overall, older breast cancer survivors with higher perceived cognitive impairment reported poorer physical functioning, increased frailty, and poorer QoL (*p* ≤ 0.001). Poorer visuospatial working memory and sustained attention exhibited increased frailty (*p* ≤ 0.001–0.01); while poorer sustained attention was associated with poorer physical function (*p* < 0.01). The findings of this study suggest a need for a thorough assessment of cognitive concerns and their associated outcomes in older breast cancer survivors.

**Abstract:**

Background: Older cancer survivors in general are at greater risk for cancer-related cognitive impairment (CRCI), yet few studies have explored its association with health outcomes. This study examined the association between subjective and objective measures of cognitive function and physical function, frailty, and quality of life (QoL) among older breast cancer survivors. Materials and Methods: Older breast cancer survivors who reported cognitive concerns completed surveys on patient-reported cognitive function, physical function, frailty, and QoL as well as objective tests of visuospatial working memory and sustained attention. Data were analyzed using descriptive statistics and separate linear regression models. Results: A total of 219 female breast cancer survivors completed the study. Perceived cognitive abilities were associated with better physical function, frailty, and QoL (*p* ≤ 0.001) while cognitive concerns were negatively related with these metrics (*p* ≤ 0.001). Poorer visuospatial working memory and sustained attention were linked to increased frailty (*p* ≤ 0.001–0.01), whereas poorer sustained attention was associated with poorer physical function (*p* < 0.01). Conclusions: Older breast cancer survivors with perceived cognitive impairment and poorer cognitive performance reported poorer physical functioning, increased frailty, and poorer QoL. These findings underscore the importance of assessing cognitive concerns and their associated outcomes in older breast cancer survivors.

## 1. Introduction

There are an estimated 18 million cancer survivors in the United States (U.S). Breast cancer is the most prevalent cancer in females, making up approximately 22% of all cancer survivors in the U.S [1]. Furthermore, approximately two-thirds of these survivors are aged 65 years or older [1]. As the population ages, the number of older adults diagnosed and treated for cancer will continue to increase [2]. By 2040, it is projected that 74% of all cancers will occur in older adults, with the greatest increase expected in adults aged 75 years and older [2]. Despite this projected growth, research is still limited in addressing the long-term effects of cancer and its treatment among older cancer survivors. 

Older cancer survivors often encounter common age-related concerns which may be compounded by the effects of the cancer and its treatment [3]. Of those concerns, cancer-related cognitive impairment (CRCI) is frequently reported [4]. There is also evidence that suggests that cancer and its treatment may accelerate the rate of cognitive aging processes in cancer survivors [3,5]. In fact, researchers have reported that up to 75% of breast cancer survivors [6] reported significant cognitive concerns after cancer treatment has ended. Older breast cancer survivors may be at greater risk for CRCI due to accelerated aging as well as lower cognitive reserve prior to treatment, in addition to increased comorbidities [7,8,9]. Although they are potentially at greater risk, few studies have focused on assessing both subjective and objective indicators of CRCI and their associated health outcomes in older cancer survivors. 

Assessing cognitive health is complex, especially among cancer survivors [10]. Recommendations from the International Cancer and Cognition Task Force have focused on the use of objective neuropsychological tests to assess deficits primarily in attention, memory, and processing speed [11,12]. While objective performance tests are often preferred, subjective or self-reported assessments can assist in identifying the subtle changes noted after cancer and cancer treatment [13]. Thus, both subjective and objective measurements of cognitive performance are valuable in understanding CRCI and its impact on health outcomes that are important for maintaining independence in older cancer survivors [14]. 

Cancer survivors in general are at risk for poor health outcomes including declines in physical function, increased frailty, and poor quality of life. Older cancer survivors with CRCI may be at even greater risk and often report poorer physical functioning and quality of life in the long-term [15,16]. Physical functioning is crucial for maintaining independent living and decreased physical functioning and frailty have been associated with increased hospitalization, a need for long-term care services, higher fall risk, poorer quality of life, and premature death in older adults [17,18]. However, previous research has neglected to examine these important outcomes in older breast cancer survivors with CRCI [19]. 

Therefore, to fully characterize CRCI, we employed both subjective reports and objective cognitive function assessments to examine whether cognitive function is associated with important health outcomes in older cancer survivors including physical function, frailty, and quality of life (QoL). As the population of cancer survivors continues to age, this research will be crucial for understanding the specific needs of older breast cancer survivors.

## 2. Materials and Methods

This study encompasses one of the primary aims of a larger investigation examining factors associated with CRCI in breast and colorectal cancer survivors (ClinicalTrials.gov identifier: NCT04611620). Breast and colorectal cancer survivors were recruited nationally via Institutional Review Board-approved social media (i.e., Facebook) and online cancer-affiliated resource sites (e.g., Pink Ribbon Connection, Dr. Susan Love Foundation, Colorectal Cancer Alliance, etc.). 

In this parent study, cancer survivors were enrolled if they indicated they were 21 years of age or older, diagnosed with breast or colorectal cancer regardless of the stage at diagnosis, at least 6 months post-adjuvant or neo-adjuvant therapy (except for current aromatase inhibitors or tamoxifen use for breast cancer survivors), and identified as having cancer-related cognitive concerns (e.g., working memory issues, problems with attention, etc.). We purposely examined responses from those that identified themselves as 60 years of age and older and who completed the online surveys and objective cognitive tests which assessed visuospatial working memory and sustained attention/processing speed. All activities were approved by both the NCI-designated Cancer Center Scientific Review Committee and the Institutional Review Board at the investigators’ university.

### 2.1. Procedures

Potential participants, recruited through social media or cancer-affiliated sites, were sent flyers and links regarding the study. Interested individuals were asked to complete an eligibility checklist. Those deemed eligible were directed to complete the REDCap^®^ online written informed consent and HIPAA forms prior to administration of the study surveys. Once informed consent was obtained, participants were assigned unique identifiers within the system. Then, a secure link to a battery of survey questions was provided using the REDCap^®^ platform. If the surveys were completed successfully, participants were sent a link and instructions via email to complete the online neuropsychological tests. Data were collected online from November 2020 to January 2021. 

### 2.2. Measures

Survivors completed surveys which included demographic information, medical, and treatment characteristics, patient-reported cognitive function (PROMIS Cognitive Concerns and Cognitive Abilities), physical function (PF-10), frailty (Tilburg Frailty Indicator-TFI), and quality of life (PROMIS Meaning and Purpose). In addition, participants completed online objective neuropsychological cognitive tests of visuospatial working memory, sustained attention, and processing speed. 

**Demographic and clinical information:** Sociodemographic data such as age, sex, race, marital status, highest level of education completed, current work status, and income were gathered through self-report. The participants also provided medical information such as the stage of cancer at diagnosis (I, II, III, IV, or unknown), the types of treatment they have received (chemotherapy, surgery, or radiation), and other illnesses aside from cancer. Using the modified Charlson Comorbidity Index (CCI) formula, comorbidity was determined based on the persons’ age and existing medical conditions aside from cancer [20].

**Subjective Cognitive Function:** The PROMIS version 1.0 short-form subscales of Cognitive Abilities and Cognitive Concerns, each containing 8 items, were used to assess subjective cognitive function. The Cognitive Abilities items target positive self-assessments of cognitive functioning, such as “My memory has been as good as usual” and “I have been able to concentrate.” Higher scores indicate higher cognitive ability. The Cognitive Concerns items are worded negatively and express concerns in the same areas. Two examples are “My thinking has been slow” and “I have had trouble shifting back and forth between different activities that require thinking.” Higher scores indicate higher cognitive concerns. Items are summed to create a total score for each subscale. These relatively short scales were derived from an intensive item analysis using Item Response Theory and qualitative analysis to produce short but reliable measures [21]. 

**Objective (Performance) Neuropsychological Assessment:** A neuropsychological assessment was conducted online using CANTAB (https://cambridgecognition.com/), Cambridge Cognition^®^ (Cambridge Cognition Ltd., Cambridge, UK), a reliable and valid program used in several clinical populations including cancer survivors to assess cognitive performance [22]. The specific cognitive domains assessed were selected from CANTAB, Cambridge Cognition^®^ as common areas of concern for cancer survivors. For this study, we assessed the following cognitive function domains using specific CANTAB, Cambridge Cognition^®^ programs: (1) visuospatial working memory capacity measured by the CANTAB^®^–SSPFSL and (2) sustained attention/processing speed via CANTAB–rapid visual information processing (RVPA). Higher scores on these tests indicate better cognitive performance. 

**Physical Functioning/Physical Limitations:** The 10-item Physical Functioning Scale (PF-10) is a subscale of the Medical Outcomes Study 36-item Short Form Health Survey (SF-36). The SF-36 and its subscales, including the PF-10, have been established as a comprehensive measure of general health that has shown reliability and validity in various populations, including cancer patients [23]. The PF-10 measures the participants perceived limitations in physical functioning during the past 4 weeks on a 3-point scale (“yes, limited a lot,” “yes, limited a little,” and “no, not limited at all”), using the original 0–100 scoring with higher scores indicating less limitation or disability [24]. Descriptively, previous research in chronically ill patients has categorized scores of the PF-10 into tertiles with a score of 0–33 defined as ‘low’, 34–66 as ‘intermediate’, and 67–100 as ‘good’ [25].

**Frailty: The Tilburg Frailty Indicator** (TFI) is a 15-item questionnaire based on a multidimensional approach to frailty, assessing physical, psychologic, and social aspects of human functioning [26,27]. The score on total frailty ranges from 0 to 15, and the ranges of the scores on physical, psychological, and social frailty components are 0 to 8, 0 to 4, and 0 to 3, respectively. Higher scores indicate higher levels of frailty. An individual is considered frail if the total TFI-score is five or higher, and for the individual subscales, three or higher in physical and two or higher for psychological and social scores indicate frailty in those components. 

**Quality of Life—Meaning and Purpose**: Quality of life was assessed using the PROMIS^®^ Meaning and Purpose short form 4a measure [28]. The PROMIS^®^ measures are standardized, reliable, and valid measures. This 4-item scale uses 5-point Likert scales from 0 to 4, with higher scores indicating better quality of life or more meaning and purpose in life. The raw score can be converted to a t-score, in which a score of 50 is considered average for the general population [28].

### 2.3. Data Analysis

Descriptive statistics were used to summarize both subject characteristics and the main variables in the study. Separate linear regression models were conducted examining the relationship between applied cognitive abilities, applied cognitive general concerns, spatial span, or sustained attention and each outcome (physical function, frailty, and QoL), controlling for the known covariates including age, stage of cancer, and CCI score. The type of treatment received, specifically chemotherapy and radiation, was strongly correlated with stage of cancer. Thus, type of treatment was not included in the models to prevent multicollinearity issues with cancer stage. Using single predictor models, the effect of treatment (received chemotherapy vs. did not receive chemotherapy and received radiation vs did not receive radiation) were not significantly associated with the outcomes. We presented F-statistic, beta weights, r^2^ and significance level for each outcome including physical function, frailty, and quality of life. All analyses were performed using SAS version 9.4 (SAS Institute, Inc., Cary, NC, USA). The significance level was set at *p* ≤ 0.05 for all tests.

## 3. Results

### 3.1. Participants Characteristics

A total of 219 female breast cancer survivors completed the study. The study participants were on average 65 years of age. Table 1 displays demographic and medical information. Briefly, the majority of participants were White (94%), married or living with a partner (71%), and had a minimum of a four-year college education (62%). In addition, the majority of the cancer survivors underwent surgery (99%) and received radiation (69%) and chemotherapy (86%) as part of their adjuvant therapy. 

Table 2 displays the mean, standard deviation, and range of all variables assessed during the study. Based on published norms, the majority were considered frail (74%, TFI ≥ 5), with 60% physical, 74% psychological, and 34% social components above established cut scores. In addition, mean scores for physical function were 64.13, indicating intermediate levels of physical functioning and a raw score of 14.2, SD = 4.09 (or 46.1 t-score, SE = 3.6) on the PROMIS quality of life meaning and purpose scale, which is considered just below the U.S. average [29]. In the following section we outline each of these models and significant predictor variables for each outcome (Table 3 and Table 4).

### 3.2. Physical Functioning

After adjusting for age at survey, cancer stage, and CCI score, applied cognitive abilities and applied cognitive general concerns were both significantly related to physical functioning in separate regression models (*p* ≤ 0.001). As demonstrated in Table 3, higher cognitive abilities were positively related to physical function whereas increased cognitive concerns were negatively related to physical function. In addition, sustained attention and speed of processing had a significant effect on physical functioning with better cognitive performance relating significantly to better physical functioning (*p* < 0.01) after controlling for covariates (See Table 4). However, visuospatial working memory was not significantly associated with physical functioning.

### 3.3. Frailty

After controlling for covariates, applied cognitive abilities and applied cognitive general concerns were significantly related to overall frailty (*p* ≤ 0.001) in separate regression models. Higher self-reported cognitive abilities were associated with less frailty, whereas greater cognitive concerns were associated with increased frailty (Table 3). Visuospatial working memory was also significantly related to overall frailty (*p* ≤ 0.001), with better performance on a working memory task associated with lower frailty scores. In addition, sustained attention/speed of processing working memory had a significant effect on overall frailty (*p* < 0.01), with those cancer survivors with higher cognitive performance exhibiting less frailty.

### 3.4. Quality of Life

Applied cognitive abilities and applied cognitive general concerns were significantly related to quality of life (*p* ≤ 0.001) in separate regression models. Higher self-reported cognitive abilities were associated with better quality of life in these cancer survivors. Increased cognitive concerns, on the other hand, were associated with poorer quality of life (See Table 3). Table 4 shows that the objective measures of visuospatial working memory and sustained attention/speed of processing were not significantly related to quality of life.

## 4. Discussion

This study was designed to fully examine both subjective and objective CRCI and their relationships to important health outcomes for older breast cancer survivors. One of the major findings of this research was that the subjective or self-reported measures of cognitive function were associated with all three health outcomes, including physical function, frailty, and quality of life. Specifically, the PROMIS measures of cognitive abilities and cognitive concerns, which have been validated in cancer survivors to tap into different dimensions of perceived cognitive function [30,31,32], identified important outcomes for older cancer survivors. These findings support the use of patient reported outcomes (PROs) such as the PROMIS measures when investigating CRCI in cancer survivors [13]. In addition, this work provides a direct linkage between subjective CRCI and physical functioning, frailty, and quality of life outcomes, suggesting that identifying CRCI early may be important for preventing these adverse outcomes.

It should be noted that each of the outcomes examined in this study is critical for maintaining independence and well-being among older cancer survivors. Physical functioning, as measured by the PF-10, was significantly correlated with subjective cognitive abilities and concerns as well as sustained attention/speed of processing, but not with visuospatial working memory in our cohort. Similar results have been noted regarding the relationship of subjective CRCI and physical functioning in older healthy adults [33] as well as in older cancer survivors [15,34]. Crouch et al. noted that perceived attention function was significantly related to physical functioning in 335 older cancer survivors [15]. However, in that same study and others, it was noted that objective measures of cognitive function were not related to physical function [15,35]. These authors suggest that objective cognitive assessments may not be sufficient to identify the subtle cognitive changes incurred after treatment, and thus fail to establish these important relationships.

The importance of both cognitive and physical health has grown significantly in recent years. Many intervention studies have examined the impact of physical activity or exercise on CRCI in cancer survivors [36]. Although, the exact underlying mechanisms remain unclear, some authors suggest that the protective effect of physical activity on cognitive function is linked to the preservation of cellular function, reduction in oxidative stress, and reduction in proinflammatory cytokines [37]. Other studies suggest that physical activity decreases psychological factors such as anxiety, fatigue, and depression, which contributes to enhancing cognitive function [36]. Regardless, work is still needed to promote physical activity in cancer survivors. In a recent observational study, Salerno et al. [38] found that more than 60% of cancer survivors who are 6 months post-treatment do not meet the recommended physical activity requirements. These findings underscore the importance of promoting physical activity to enhance physical functioning and potentially improve or stave off cognitive decline in older cancer survivors.

Frailty, as measured by the self-reported TFI scale, was quite high in the sample. Overall, 73% of the survivors were identified as frail, which was rather surprising given the relatively young age (M = 65 years old) and composition of the sample (e.g., majority with a college degree, married or partnered, etc.). It is plausible that the higher scores on the TFI may have been due to added psychological impact of COVID-19 for cancer survivors during the study period [39]. The emotional and psychological impact of COVID-19, including anxiety and uncertainty of the future, were universal concerns at the time; however, researchers identified that cancer survivors were particularly vulnerable [39]. Future research may benefit from collecting information regarding COVID-19 concerns as confounding CRCI and/or self-reported frailty or alternatively utilize an objective measure to assess frailty in older cancer survivors [40].

We found that all measures of CRCI (multiple subjective and objective measures) were associated with frailty. Cognitive impairment and frailty have been shown to be distinct but interrelated conditions that are associated with changes in function and are thought to be caused by cancer and/or cancer treatment. Both cognitive impairment and frailty are major concerns in our aging population, as they predispose older adults to disabilities, a poor quality of life, and mortality [38]. In the older adult population, frailty is linked with decreased global cognitive function, as well as a decline in specific functions such as processing speed, working memory, and sustained attention [41]. In a sample of older long-term breast cancer survivors, CRCI was associated with higher frailty scores [42]. Similarly, Magnuson documented that longitudinal decline in subjective and objective measures of attention and memory were associated with increased frailty from pre-treatment to post-treatment in breast cancer survivors aged 50 years and older [43]. Frailty is also a significant predictor of other health outcomes, especially in older cancer survivors. Cancer survivors identified as frail or pre-fail are more likely to incur treatment toxicities, experience increased hospitalizations, report increased cancer-related symptoms (pain, neuropathy, and fatigue), and are at increased risk of all-cause mortality [44,45,46]. Taken together, more research is needed to explore the relationships between cognitive impairment and frailty and the accelerated aging associated with cancer and cancer treatment in older cancer survivors.

In this study, we found that subjective CRCI was associated with QoL in cancer survivors. Similarly, previous studies have documented that subjective CRCI is associated with QoL in breast cancer survivors [34]. The association between subjective cognitive impairment and quality of life has been reported in both older and younger cancer survivors [47,48]. Researchers have also identified that breast cancer survivors with higher psychoneurological symptoms and lower self-reported cognitive function prior to treatment have lower breast-cancer specific QoL at 12 and 24 months after systemic therapy [48]. One systematic review in older adult cancer survivors showed that both physically and cognitively frail older cancer patients who reported worse symptoms before cancer treatments reported worse symptom toxicity and poorer QoL after cancer treatments, compared to non-frail cancer patients [49]. Thus, assessment of cognitive function prior to treatment is essential to evaluate the need for additional support in those who already have high baseline cognitive impairment. Although subjective CRCI was related to QoL, we did not find significant relationships between objective cognitive performance tests and QoL. Previous research regarding objective measures of CRCI and QoL have been mixed [15,48,50]. Researchers have suggested that findings may vary based on the QoL instrument used [15]. More work is needed to understand the relationship between CRCI and QoL, especially when utilizing objective measures to determine cognitive impairment.

## 5. Strengths and Limitations

The strengths of this study include the large national sample of cancer survivors using both subjective and objective measures to assess cognitive function. In addition, this study focused on a larger number of outcomes including measures of physical function, frailty, and QoL, and controlled for potential confounding factors (e.g., age, stage of disease, etc.). However, the study was limited by the mostly White and female sample population, and thus does not represent all older breast cancer survivors. In addition, the cross-sectional design limits interpretation to associations between variables versus establishing a causal effect. Future longitudinal studies with more diverse participants will be vital in exploring the role of sociodemographic characteristics in the relationship between cognitive impairment, physical function, frailty, and quality of life across the cancer care trajectory. In addition, future studies should consider objective measures of physical functioning and frailty to determine if these associations remain.

## 6. Conclusions

This study used both subjective and objective measures of CRCI to explore the association with health outcomes (physical function, frailty, and QoL) which are salient for determining the long-term well-being of older cancer survivors. By emphasizing the use of subjective measures in assessing CRCI and connecting these assessments to significant health outcomes, this study highlighted the importance of early identification and management of CRCI to mitigate adverse effects such as declining physical functioning, increased frailty, and diminished quality of life in older cancer survivors. Furthermore, we demonstrated the relationship between cognitive function and frailty, further supporting the hypothesis that cancer and its treatment may accelerate cognitive aging and lead to adverse outcomes in older cancer survivors. Early identification and management of CRCI may be important to prevent further untoward effects, including decline in physical functioning, increased frailty, and poorer quality of life in older cancer survivors.

## Figures and Tables

**Table 1:** Characteristics of the sample (N = 219).

Characteristics	N (%)
**Age**	
Mean	65.3
Median	64.2
Range	59.0–88.0
**Race**	
White	205 (93.6%)
Black	8 (3.7%)
Other	3 (1.4%)
More than one Race	2 (0.9%)
Unknown/Prefer not to Answer	1 (0.5%)
**Marital Status**	
Single	14 (6.4%)
Married/Living with Partner	155 (70.8%)
Divorced	30 (13.7%)
Widowed	18 (8.2%)
Other/Prefer not to Answer	2 (0.9%)
**Highest level of education completed**	
High School Graduate	22 (10.1%)
Associate’s Degree/Some College	62 (28.3%)
Undergraduate/Bachelor’s Degree or Equivalent	68 (31.1%)
Master’s Degree or Equivalent	50 (22.8%)
PhD or Equivalent	17 (7.8%)
**Current Work Status**	
Full-time (>35 h/week)	52 (23.7%)
Part-time (<20 h/week)	23 (10.5%)
Retired/Homemaker/Unemployed	139 (63.5%)
Other/Prefer not to Answer	5 (2.3%)
**Income category**	
<USD 15,000	5 (2.3%)
USD 15,001 to USD 30,000	19 (8.7%)
USD 30,001 to USD 50,000	41 (18.7%)
USD 50,001 to USD 75,000	42 (19.2%)
USD 75,000 to USD 100,000	33 (15.1%)
USD 100,001 to USD 150,000	28 (12.8%)
USD 150,001 to USD 200,000	10 (4.6%)
>USD 200,000	11 (5.0%)
Unknown/Prefer not to Answer	30 (13.7%)
**Stage of cancer at diagnosis**	
Stage I	68 (31.1%)
Stage II	88 (40.2%)
Stage III	46 (21.0%)
Stage IV	6 (2.7%)
Unsure/Prefer not to Answer	11 (5.0%)
**Received chemotherapy**	
No	30 (13.7%)
Yes	189 (86.3%)
**Underwent surgery**	
No	2 (0.9%)
Yes	217 (99.1%)
**Received radiation therapy**	
No	67 (30.6%)
Yes	152 (69.4%)
**Years since diagnosis**	
Mean	8.2
Median	6.2
Range	0.3–34.0

**Table 2:** Means and standard deviations of main variables.

Characteristic	N	Mean (SD)	Actual Range
**Cognitive Functioning**			
Applied Cognitive General Concerns	219	25.51 (7.34)	(8.0, 40.0)
Applied Cognitive Abilities	219	21.96 (6.85)	(8.0, 38.0)
Sustained Attention: CANTAB-RVPA	151	0.91 (0.05)	(0.71, 0.99)
Spatial Span: CANTAB-SSPFSL	153	5.95 (1.24)	(4.0, 9.0)
Physical Functioning	219	64.13 (27.10)	(0.0, 100.0)
Quality of Life (Meaning/Purpose)	219	14.20 (4.09)	(4.0, 20.0)
**Frailty**			
Overall TFI score	219	6.43 (2.86)	(0.0,14.0)
Physical component score	219	3.04 (1.92)	(0.0, 8.0)
Psychological component score	219	2.14 (1.13)	(0.0, 4.0)
Social component score	219	1.25 (0.77)	(0.0, 3.0)
**N (%) categorized as frail**			
Overall TFI score ≥ 5	219	161 (73.52%)	
Physical component score ≥ 3	219	131 (59.82%)	
Psychological component score ≥ 2	219	161 (73.52%)	
Social component score ≥ 2	219	75 (34.25%)	

**Table 3:** Association between subjective cognitive function and physical function, frailty, and quality of life, controlling for covariates.

Predictor Variable	Physical Function β (SE)	Frailty β (SE)	Quality of Life β (SE)
**Cognitive Abilities**	1.30 (0.25) ***	−0.23 (0.02) ***	0.30 (0.04) ***
Age	0.77 (0.37) *	−0.06 (0.03)	0.01 (0.05)
Cancer Stage 2 ^1^	−2.33 (3.93)	−0.63 (0.36)	1.52 (0.57) **
Cancer Stage 3 ^1^	−9.66 (4.65) *	0.31 (0.43)	1.15 (0.67)
Cancer Stage 4 ^1^	−10.65 (10.37)	−0.04 (0.96)	−0.85 (1.50)
Cancer Stage Unknown ^1^	−4.88 (7.94)	−0.59 (0.74)	0.68 (1.15)
Charlson Comorbidity Index	−6.95 (1.82) ***	0.56 (0.17) ***	−0.10 (0.26)
F	8.53 ***	20.05 ***	12.14 ***
R^2^	22.1	40.0	28.7
Adjusted R^2^	19.5	38.0	26.3
N	219	219	219
**Cognitive Concerns**	−1.10 (0.24) ***	0.19 (0.02) ***	−0.18 (0.04) ***
Age	0.81 (0.38) *	−0.06 (0.04)	0.05 (0.06)
Cancer Stage 2 ^1^	−5.01 (4.02)	−0.16 (0.38)	1.08 (0.63)
Cancer Stage 3 ^1^	−11.42 (4.71) *	0.62 (0.45)	0.79 (0.73)
Cancer Stage 4 ^1^	−10.89 (10.51)	−0.00 (1.01)	−1.07 (1.64)
Cancer Stage Unknown ^1^	−6.84 (8.05)	−0.24 (0.77)	0.32 (1.26)
Charlson Comorbidity Index	−7.90 (1.83) ***	0.73 (0.17) ***	−0.34 (0.29)
F	7.55 ***	15.65 ***	5.17 ***
R^2^	20.0	34.2	14.7
Adjusted R^2^	17.4	32.0	11.8
N	219	219	219

* *p* < 0.05, ** *p* < 0.01, *** *p* ≤ 0.001, ^1^ Reference Group: Cancer Stage 1.

**Table 4:** Association between objective cognitive function and physical function and frailty, controlling for covariates.

Predictor Variable	Physical Function β (SE)	Frailty β (SE)	Quality of Life β (SE)
**Working Memory**	2.66 (1.49)	−0.54 (0.16) ***	0.30 (0.26)
Age	1.15 (0.40) **	−0.13 (0.04) **	0.00 (0.07)
Cancer Stage 2 ^1^	1.01 (4.26)	−0.51 (0.47)	1.48 (0.73) *
Cancer Stage 3 ^1^	−15.15 (5.63) **	0.46 (0.62)	1.85 (0.97)
Cancer Stage 4 ^1^	−16.51 (9.84)	0.76 (1.08)	−1.87 (1.69)
Cancer Stage Unknown ^1^	−7.15 (8.27)	−0.89 (0.91)	0.87 (1.42)
Charlson Comorbidity Index	−8.13 (2.22) ***	0.54 (0.24) *	0.05 (0.38)
F	4.76 ***	3.84 ***	1.42
R^2^	18.7	15.6	6.4
Adjusted R^2^	14.8	11.6	1.9
N	153	153	153
**Sustained Attention/Processing Speed**	124.52 (40.61) **	−14.41 (4.56) **	12.78 (7.06)
Age	1.38 (0.39) ***	−0.16 (0.04) ***	0.04 (0.07)
Cancer Stage 2 ^1^	1.67 (4.17)	−0.57 (0.47)	1.54 (0.72) *
Cancer Stage 3 ^1^	−16.19 (5.43) **	0.68 (0.61)	1.65 (0.94)
Cancer Stage 4 ^1^	−16.33 (9.48)	0.92 (1.06)	−1.94 (1.65)
Cancer Stage Unknown ^1^	−9.47 (8.04)	−0.55 (0.90)	0.54 (1.40)
Charlson Comorbidity Index	−6.74 (2.19) **	0.38 (0.25)	0.18 (0.38)
F	6.14 ***	4.11 ***	1.84
R^2^	23.1	16.8	8.3
Adjusted R^2^	19.4	12.7	3.8
N	151	151	151

* *p* < 0.05, ** *p* < 0.01, *** *p* ≤ 0.001, ^1^ Reference Group: Cancer Stage 1.

## Data Availability

The datasets presented in this article are not readily available due to technical limitations. Requests to access the datasets should be directed to Dr. Diane Von Ah (vonah.1@osu.edu).

## References

[1] American Cancer Society (2022). Cancer Treatment & Survivorship Facts & Figures 2022–2024.

[2] Weir H.K., Thompson T.D., Stewart S.L., White M.C. (2021). Cancer Incidence Projections in the United States between 2015 and 2050. Prev. Chronic Dis..

[3] Ahles T.A., Root J.C. (2018). Cognitive Effects of Cancer and Cancer Treatments. Annu. Rev. Clin. Psychol..

[4] Lange M., Licaj I., Clarisse B., Humbert X., Grellard J.M., Tron L., Joly F. (2019). Cognitive complaints in cancer survivors and expectations for support: Results from a web-based survey. Cancer Med..

[5] Guida J.L., Ahles T.A., Belsky D., Campisi J., Cohen H.J., DeGregori J., Fuldner R., Ferrucci L., Gallicchio L., Gavrilov L. (2019). Measuring Aging and Identifying Aging Phenotypes in Cancer Survivors. J. Natl. Cancer Inst..

[6] Boscher C., Joly F., Clarisse B., Humbert X., Grellard J.M., Binarelli G., Tron L., Licaj I., Lange M. (2020). Perceived Cognitive Impairment in Breast Cancer Survivors and Its Relationships with Psychological Factors. Cancers.

[7] Crouch A., Champion V.L., Unverzagt F.W., Pressler S.J., Huber L., Moser L.R., Cella D., Von Ah D. (2022). Cognitive dysfunction prevalence and associated factors in older breast cancer survivors. J. Geriatr. Oncol..

[8] Ahles T.A., Root J.C., Ryan E.L. (2012). Cancer- and cancer treatment-associated cognitive change: An update on the state of the science. J. Clin. Oncol..

[9] Mandelblatt J.S., Small B.J., Luta G., Hurria A., Jim H., McDonald B.C., Graham D., Zhou X., Clapp J., Zhai W. (2018). Cancer-Related Cognitive Outcomes among Older Breast Cancer Survivors in the Thinking and Living with Cancer Study. J. Clin. Oncol..

[10] Orszaghova Z., Mego M., Chovanec M. (2021). Long-Term Cognitive Dysfunction in Cancer Survivors. Front. Mol. Biosci..

[11] Joly F., Giffard B., Rigal O., De Ruiter M.B., Small B.J., Dubois M., LeFel J., Schagen S.B., Ahles T.A., Wefel J.S. (2015). Impact of Cancer and Its Treatments on Cognitive Function: Advances in Research From the Paris International Cognition and Cancer Task Force Symposium and Update Since 2012. J. Pain Symptom Manag..

[12] Wefel J.S., Vardy J., Ahles T., Schagen S.B. (2011). International Cognition and Cancer Task Force recommendations to harmonise studies of cognitive function in patients with cancer. Lancet Oncol..

[13] Bray V.J., Dhillon H.M., Vardy J.L. (2018). Systematic review of self-reported cognitive function in cancer patients following chemotherapy treatment. J. Cancer Surviv..

[14] Crouch A., Champion V., Von Ah D. (2022). Cognitive Dysfunction in Older Breast Cancer Survivors. Cancer Nurs..

[15] Crouch A., Champion V.L., Von Ah D. (2021). Comorbidity, cognitive dysfunction, physical functioning, and quality of life in older breast cancer survivors. Support. Care Cancer.

[16] Kent E.E., Ambs A., Mitchell S.A., Clauser S.B., Smith A.W., Hays R.D. (2014). Health-related quality of life in older adult survivors of selected cancers: Data from the SEER-MHOS linkage. Cancer.

[17] Beswick A.D., Rees K., Dieppe P., Ayis S., Gooberman-Hill R., Horwood J., Ebrahim S. (2008). Complex interventions to improve physical function and maintain independent living in elderly people: A systematic review and meta-analysis. Lancet.

[18] Brown R.T., Diaz-Ramirez L.G., Boscardin W.J., Lee S.J., Steinman M.A. (2017). Functional Impairment and Decline in Middle Age. Ann. Intern. Med..

[19] Jia M., Zhang X., Wei L., Gao J. (2021). Measurement, outcomes and interventions of cognitive function after breast cancer treatment: A narrative review. Asia Pac. J. Clin. Oncol..

[20] National Cancer Institute NCI Comorbidity Index Overview. https://healthcaredelivery.cancer.gov/seermedicare/considerations/comorbidity.html.

[21] Cella D., Riley W., Stone A., Rothrock N., Reeve B., Yount S., Amtmann D., Bode R., Buysse D., Choi S. (2010). The Patient-Reported Outcomes Measurement Information System (PROMIS) developed and tested its first wave of adult self-reported health outcome item banks: 2005–2008. J. Clin. Epidemiol..

[22] CANTAB^®^ (2023). Cognitive Assessment Software.

[23] Hays R.D., Sherbourne C.D., Mazel R.M. (1993). The rand 36-item Health Survey 1.0. Health Econ..

[24] Von Ah D., Russell K.M., Storniolo A.M., Carpenter J.S. (2009). Cognitive Dysfunction and Its Relationship to Quality of Life in Breast Cancer Survivors. Oncol. Nurs. Forum..

[25] van Loon I., Hamaker M.E., Boereboom F.T.J., Grooteman M.P.C., Blankestijn P.J., van den Dorpel R.M.A., Nubé M.J., Ter Wee P.M., Verhaar M.C., Bots M.L. (2017). A closer look at the trajectory of physical functioning in chronic hemodialysis. Age Ageing.

[26] Gobbens R.J., van Assen M.A., Luijkx K.G., Wijnen-Sponselee M.T., Schols J.M. (2010). The Tilburg Frailty Indicator: Psychometric properties. J. Am. Med. Dir. Assoc..

[27] Gobbens R.J., Schols J.M., van Assen M.A. (2017). Exploring the efficiency of the Tilburg Frailty Indicator: A review. Clin. Interv. Aging.

[28] Salsman J.M., Schalet B.D., Park C.L., George L., Steger M.F., Hahn E.A., Snyder M.A., Cella D. (2020). Assessing meaning & purpose in life: Development and validation of an item bank and short forms for the NIH Promis^®^. Qual. Life Res..

[29] PROMIS (2021). PROMIS Meaning and Purpose Scoring Manual.

[30] Henneghan A.M., Van Dyk K., Zhou X., Moore R.C., Root J.C., Ahles T.A., Nakamura Z.M., Mandeblatt J., Ganz P.A. (2023). Validating the PROMIS cognitive function short form in cancer survivors. Breast Cancer Res. Treat..

[31] Lai J.S., Butt Z., Wagner L., Sweet J.J., Beaumont J.L., Vardy J., Jacobsen P.B., Shapiro P.J., Jacobs S.R., Cella D. (2009). Evaluating the dimensionality of perceived cognitive function. J. Pain Symptom Manag..

[32] Lai J.S., Wagner L.I., Jacobsen P.B., Cella D. (2014). Self-reported cognitive concerns and abilities: Two sides of one coin?. Psychooncology.

[33] Cosentino S., Devanand D., Gurland B. (2018). A Link between Subjective Perceptions of Memory and Physical Function: Implications for Subjective Cognitive Decline. J. Alzheimers Dis..

[34] Mandelblatt J.S., Zhai W., Ahn J., Small B.J., Ahles T.A., Carroll J.E., Denduluri N., Dilawari A., Extermann M., Graham D. (2020). Symptom burden among older breast cancer survivors: The Thinking and Living With Cancer (TLC) study. Cancer.

[35] Lange M., Giffard B., Noal S., Rigal O., Kurtz J.E., Heutte N., Levy C., Allouache D., Rieux C., Le Fel J. (2014). Baseline cognitive functions among elderly patients with localised breast cancer. Eur. J. Cancer.

[36] Myers J.S., Erickson K.I., Sereika S.M., Bender C.M. (2018). Exercise as an Intervention to Mitigate Decreased Cognitive Function from Cancer and Cancer Treatment. Cancer Nurs..

[37] Campbell K.L., Winters-Stone K.M., Wiskemann J., May A.M., Schwartz A.L., Courneya K.S., Zucker D.S., Matthews C.E., Ligibel J.A., Gerber L.H. (2019). Exercise Guidelines for Cancer Survivors: Consensus Statement from International Multidisciplinary Roundtable. Med. Sci. Sports Exerc..

[38] Salerno E.A., Culakova E., Kleckner A.S., Heckler C.E., Lin P.J., Matthews C.E., Conlin A., Weiselberg L., Mitchell J., Mustian K.M. (2021). Physical Activity Patterns and Relationships With Cognitive Function in Patients With Breast Cancer Before, During, and After Chemotherapy in a Prospective, Nationwide Study. J. Clin. Oncol..

[39] Muls A., Georgopoulou S., Hainsworth E., Hartley B., O’Gara G., Stapleton S., Cruickshank S. (2022). The psychosocial and emotional experiences of cancer patients during the COVID-19 pandemic: A systematic review. Semin. Oncol..

[40] Bieniek J., Wilczyński K., Szewieczek J. (2016). Fried frailty phenotype assessment components as applied to geriatric inpatients. Clin. Interv. Aging.

[41] Li C., Ge S., Yin Y., Tian C., Mei Y., Han P. (2023). Frailty is associated with worse cognitive functioning in older adults. Front. Psychiatry.

[42] Ahles T.A., Schofield E., Li Y., Ryan E., Root J.C., Patel S.K., McNeal K., Gaynor A., Tan H., Katheria V. (2022). Relationship between cognitive functioning and frailty in older breast cancer survivors. J. Geriatr. Oncol..

[43] Magnuson A., Lei L., Gilmore N., Kleckner A.S., Lin F.V., Ferguson R., Hurria A., Wittink M.N., Esparaz B.T., Giguere J.K. (2019). Longitudinal Relationship Between Frailty and Cognition in Patients 50 Years and Older with Breast Cancer. J. Am. Geriatr. Soc..

[44] Williams G.R., Dunham L., Chang Y., Deal A.M., Pergolotti M., Lund J.L., Guerard E., Kenzik K., Muss H.B., Sanoff H.K. (2019). Geriatric Assessment Predicts Hospitalization Frequency and Long-Term Care Use in Older Adult Cancer Survivors. J. Oncol. Pract..

[45] Ethun C.G., Bilen M.A., Jani A.B., Maithel S.K., Ogan K., Master V.A. (2017). Frailty and cancer: Implications for oncology surgery, medical oncology, and radiation oncology. CA Cancer J. Clin..

[46] Zhai C., Yin L., Shen J., Dong J., Zheng Y., Pan H., Han W. (2024). Association of Frailty with Mortality in Cancer Survivors: Results from Nhanes 1999–2018. Sci. Rep..

[47] Von Ah D., Crouch A.D., Monahan P.O., Stump T.E., Unverzagt F.W., Storey S., Cohee A.A., Cella D., Champion V.L. (2021). Association of cognitive impairment and breast cancer survivorship on quality of life in younger breast cancer survivors. J Cancer Surviv..

[48] Tometich D.B., Small B.J., Carroll J.E., Zhai W., Luta G., Zhou X., Kobayashi L.C., Ahles T., Saykin A.J., Clapp J.D. (2019). Pretreatment Psychoneurological Symptoms and Their Association with Longitudinal Cognitive Function and Quality of Life in Older Breast Cancer Survivors. J. Pain Symptom Manag..

[49] Han C.J., Rosko A.E., Spakowicz D.J., Hammer M.J., Von Ah D. (2023). Associations of frailty with symptoms, and HRQOL in older cancer survivors after cancer treatments: A systematic review and meta-analyses. Qual. Life Res..

[50] Lange M., Heutte N., Rigal O., Noal S., Kurtz J.E., Levy C., Allouache D., Rieux C., Lefel J., Clarisse B. (2016). Decline in Cognitive Function in Older Adults with Early-Stage Breast Cancer after Adjuvant Treatment. Oncologist.

